# Effect of Picture-Book Reading With Additive Audio on Bilingual Preschoolers’ Prefrontal Activation: A Naturalistic Functional Near-Infrared Spectroscopy Study

**DOI:** 10.3389/fpsyg.2020.01939

**Published:** 2020-08-05

**Authors:** Chuanjiang Li, Keya Ding, Mingming Zhang, Li Zhang, Jing Zhou, Dongchuan Yu

**Affiliations:** ^1^Key Laboratory of Child Development and Learning Science of Ministry of Education, Research Center for Learning Science, Southeast University, Nanjing, China; ^2^School of Psychology, Shanghai Normal University, Shanghai, China; ^3^Faculty of Education, East China Normal University, Shanghai, China

**Keywords:** bilingual advantage, picture-book reading, story audio, English comprehension, functional near-infrared spectroscopy, prefrontal cortex

## Abstract

Acquiring a second language (L2) has the power to shape cognition and even the function and structure of the brain. Picture-book reading with additive audio (PRA) is a popular and convenient means of providing L2 exposure for non-balanced bilingual children; however, its contribution to bilingual children’s brain activity is unclear. This study conducted a rigorous bilingual word comprehension experiment and a naturalistic PRA task to explore the effect of L2 processing on brain activation among English as a foreign language (EFL) preschoolers, using functional near-infrared spectroscopy (fNIRS). We found that the two contexts of comprehending English words and bilingual switching (BS), which impose more cognitive control demands, activated the prefrontal cortex (PFC) more than did the condition of comprehending Chinese words. Furthermore, the effect of PFC activity in the condition of picture-book reading with additive English audio (English PRA) was also found to be greater than in the condition of picture-book reading with additive Chinese audio (Chinese PRA); moreover, the effect was modulated by story difficulty. Finally, a positive correlation was shown between EFL children’s English competence and PFC activation through English PRA. This study indicates that the experiences of hearing L2 auditory stories in a picture-book reading activity yielded significant changes to early bilinguals’ PFC functional for cognitive control and language processing.

## Introduction

In this era of globalization, more than half the world’s population speaks multiple languages, to some extent (Bialystok, 2017). A large body of previous studies have claimed that bilinguals outperform monolinguals in several tasks assessing executive control (Bialystok and Martin, 2004; Christoffels et al., 2013), cognitive flexibility (Adi-Japha et al., 2010), working memory (Morales et al., 2013; Blom et al., 2014), metalinguistic abilities (Barac et al., 2014), and even the incidence of Alzheimer’s disease (Craik et al., 2010). Numerous behavioral studies have summarized those positive effects of bilingualism on human cognitive development, giving rise to the hypothesis of bilingual advantage.

Furthermore, the rapid development of neuroimaging technologies – i.e., event-related potential (ERP), magnetic resonance imaging (MRI), functional near-infrared spectroscopy (fNIRS), etc., – has made it possible to depict the relative contribution of bilingual experience to the brain and mind (Kroll, 2015; García-Pentón et al., 2016). Using those new techniques, emerging studies have found some general frameworks for the bilingual effect on neural architecture from the perspectives of both functional reorganization (Abutalebi and Green, 2007; Arredondo et al., 2019b; Li et al., 2019) and structural restructuration, such as gray matter density (Mechelli et al., 2004), white matter integrity (Mohades et al., 2015), and cortical thickness (Klein et al., 2014). Thus, neuroplasticity in the bilingual brain should not come as a surprise, given such evidence of experience-based neuroplasticity (Hernandez et al., 2019) as differences in the hippocampi of a taxi driver and a bus driver (Maguire et al., 2000, 2006).

In considering the neuroplasticity of bilingualism, the basic neuromechanism of dual language processing must first be understood. According to the joint activation theory, both languages are activated when bilinguals use either, and the competition between the two may be an inherent process (Bialystok, 2011; Kroll et al., 2015). In fact, numerous studies, using techniques ranging from eye-tracking to electroencephalography (EEG), have offered evidence of activation in parallel (Spivey and Marian, 1999; Thierry and Yan, 2004, 2007; Weber and Cutler, 2004; Guo and Peng, 2006; Blumenfeld and Marian, 2007; Martin et al., 2009; Shook and Marian, 2012; Giezen et al., 2015; Chen et al., 2017). On the other hand, inhibitory control model theory (Green, 1998; Giezen et al., 2015) claims that the conflict is resolved by suppression of the non-target language competitor and that selection involves the domain-general executive control system in charge of the prefrontal cortex (PFC) (Ridderinkhof et al., 2004). That is to say, the process of dual language control boosts the function of executive control. Obviously, bilingual language control overlaps significantly with some brain regions responsible for domain-general executive control (Pliatsikas and Luk, 2016). Thus, bilingual selection and competition could explain the mechanism of bilingual neuroplasticity to some extent. The bilingual selection and control network is mainly held to encompass the dorsal lateral prefrontal cortex (DLPFC), anterior cingulate cortex (ACC), left inferior frontal gyrus (IFG), inferior parietal lobules (IPLs), and left caudate (Abutalebi and Green, 2007, 2008, 2016; Buchweitz and Prat, 2013; Cachia et al., 2017).

However, brain neuroplasticity is moderated by a series of complex language experience-based variables, such as range of ages, duration of second language (L2) acquisition, and resulting language proficiency (Luk and Bialystok, 2013; Puriæ et al., 2017; Gullifer et al., 2018; Hao et al., 2018; DeLuca et al., 2019, 2020; Legault et al., 2019). For example, the landmark framework of *Bilingual Anterior to Posterior and Subcortical Shift* (BAPSS) claims that the shift of recruitment from frontal to posterior and subcortical regions is modulated with L2 exposure (Grundy et al., 2017). Remarkably, limited but innovative studies have found that bilingual children, but not adults with more complex language experience, tend to activate greater frontal resources than monolinguals in language processing and cognitive control tasks (Kobayashi et al., 2008; Jasinska and Petitto, 2013). Childhood is a sensitive period of neuroplasticity, and in the early stage of L2 learning, there is no doubt that it is an ideal period for researchers to investigate the correlation between bilingualism and brain development.

Studies on bilingual experience and cognitive or language processing of children have gradually emerged in recent years. For example, Arredondo et al. (2017) found that early bilingualism could yield significant changes to the functional organization of schoolchildren’s PFC for non-verbal attentional control, especially within the left hemisphere, associated with normative language processing and bilingual language switching. While Moriguchi and Lertladaluck (2019) failed to find an association between L2 immersion time and brain function for cognitive shifting among 3- to 5-year-old bilinguals, suggesting insufficient L2 exposure may fail to influence neurocognitive plasticity, on the other hand, long-term early life bilingual exposure would influence children’s cortical organization for language processing. For instance, by mid-childhood, bilingual children show greater activation in posterior temporo-parietal regions that support more automated lexical recognition, while monolinguals rely on greater activity in the left frontal regions (Arredondo et al., 2019a).

Notwithstanding rising interest, most current neuroimaging studies of the bilingual effect on experience-dependent brain plasticity still focus on adults and school-aged children, rather than on young children (Barac et al., 2016). Bilingual exposure may have different effects on young children’s neural plasticity of language processing. Worse, previous bilingual control or bilingual switching (BS) studies have largely focused on language production, leaving the neural underpinnings of language comprehension blurred (Abutalebi et al., 2007). Moreover, the neural involvement of language control in production is significantly different from perception (Blanco-Elorrieta and Pylkkänen, 2016). Consequently, in this study, we first explore whether L2 understanding changes bilingual preschoolers’ prefrontal activation in a bilingual comprehension task including different cognitive control demands.

Picture-book reading is widely used in early language education globally, as an effective method for young children to accumulate language learning experience, (Whitehurst and Lonigan, 1998). Hutton et al. (2015) conducted a neuroimaging study that found that greater parent–child shared book reading increased the activation of left parietal–temporal–occipital areas supporting mental imagery and narrative comprehension. Successive studies further attribute the acceleration of children’s picture-book reading to higher cognitive brain network construction (Horowitz-Kraus et al., 2017; Hutton et al., 2017a, b). On the other hand, advances in multimedia technologies in the digital age are changing the ways in which children learn a language (Vulchanova et al., 2017), with various apps on a range of portable platforms enriching children’s ubiquitous access to story sharing and multilingual learning (Parish-Morris et al., 2013).

In particular, picture-book reading with additive audio (PRA) is becoming an important language input style for young children’s L2 learning, especially in social environments in which multiple languages are scarce (Sun et al., 2016). For instance, China – a country in which Chinese is the dominant language and English is not a community language – has a growing number of young children learning English by the PRA method. However, the effect of PRA implementation on a young English as a foreign language (EFL) learner’s brain activation is still not fully understood. Additionally, whether different degrees of story difficulty generate different effects on activation deserves further investigation. Multimedia cognitive theory believes that complex stories in multimedia learning material engage children’s cognitive load more than easy stories (Mayer, 2014). Yabe et al. (2018) have found evidence of significant increases in prefrontal blood flow in a naïve picture-book reading session when compared with a familiar session. Hence, the second aim of this study is to explore the effects of the English PRA method on children’s brain activity using a naturalistic picture-book reading neuroimaging pattern.

Functional near-infrared spectroscopy is a widely used and rapidly developing neuroimaging technique that uses non-invasive optical imaging to measure changes in hemoglobin (Hb) concentrations. Its superiority in numerous areas – e.g., safety, portability, appropriateness for young children, high temporal resolution, and insensitivity to motion noise – compared to other neuroimaging technologies makes fNIRS a primary candidate for the language tasks in the current study (Minagawa-Kawai et al., 2008).

Based on the findings and unfathomed questions mentioned above, the following hypotheses are investigated in this study. First, that English vocabulary comprehension and BS – two contexts that impose greater language control and cognitive control demands – might result in higher PFC activation than the condition of Chinese vocabulary comprehension, for EFL preschoolers. Second, that, in the PRA task, PFC activation will be higher with additive English audio than with Chinese audio. Finally, that the degree of story difficulty and children’s English proficiency will affect prefrontal activation in the English PRA task.

## Materials and Methods

### Participants

The participants of this study were 26 healthy, right-handed EFL bilingual preschoolers (male = 12; mean months old = 74.62 ± 5.68; month range = 65–84 months) from a high-quality public kindergarten in Mudanjiang, China. Two additional children were excluded because they did not continue the whole experiment owing to nervousness and/or crying and intense head motion. The recruited EFL preschoolers generally had 30–60 min of English curricular activity in kindergarten once a day and 30–60 min of extracurricular activity at an after-school English training institute twice a week. In the kindergartens, Chinese teachers (usually English majors) implemented English activities with children under the Cambridge children’s English curriculum framework. In the after-school training institute, the English learning program included interactive group activities to teach English words, phrases, songs, and reading, as selected by a native English speaker and a Chinese teacher. Together, the EFL preschoolers had spent at least 4 h per week on English learning for one and a half years. Furthermore, for all preschoolers, their family’s per capita monthly household income (>6,000 RMB) was more than twice the local per capita disposable income (Mudanjiang Bureau of Statistics, 2019); moreover, their mothers had all graduated from college or university. In other words, all participating preschoolers came from families that were above middle socioeconomic status (SES), based on local development statistics. The East China Normal University Institutional Review Board on Human Research Protection approved all aspects of the experiments.

### Language Competence Tests

The Peabody Picture Vocabulary Test – Revised (PPVT, Lu and Liu, 1998) and the Expressive Vocabulary Test (EVT, Williams, 1997) were used to assess EFL preschoolers’ Chinese receptive and expressive vocabulary, respectively. In the PPVT, children were asked to select, from a four-picture array, the one that best matched the target vocabulary; for example, children were asked to point out which of four images (a brush, a bell, a horse, and a bus) best represented the vocabulary word “bus.” In the EVT test, children were asked to provide a one-word response after being shown a colored picture and asked a prompt question (e.g., What is this animal?). Additionally, the Preschool Language Scale – fourth edition (PLS, Zimmerman et al., 2002) was conducted to measure children’s English auditory comprehension (AC) and expressive communication (EC), based on a range of interactions between the examiner and the examinee involving play material.

### Bilingual Comprehension Switch Task

Many prior verbal production studies (e.g., Abutalebi and Green, 2008; Luk et al., 2012, for a meta-analysis; Coderre et al., 2016) have found that the process of bilingualism engages more domain-general executive control neural networks to manage language processing. Based on various digital-naming and picture-naming tasks in previous language production studies, this study used the bilingual comprehension switch (BCS) task to explore EFL preschoolers’ brain activation difference when executing different cognitive control demands in three conditions of word comprehension: single Chinese comprehension (CC), single English comprehension (EC), and BS. Preschoolers were required to judge the agreement between object pictures and single language audio stimuli and make a correct choice in the CC and EC conditions. To avoid any expectation effect in the BS condition, Chinese and English audio stimuli were played to the preschoolers randomly. The 80 colored stimulus object pictures, which address name agreement and image familiarity, were adapted from Snodgrass and Vanderwart’s picture name agreement norms Snodgrass and Vanderwart (1980) and the picture naming norms for Mandarin Chinese preschool children (Wang et al., 2014).

**FIGURE 1:**
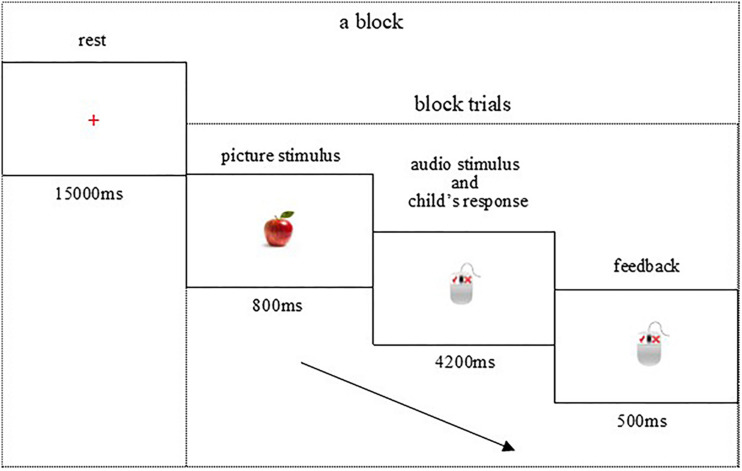
Brain imaging procedure of bilingual comprehension switch task. Each child was asked to look at the picture and listen to the English or Chinese word, and then press a button to indicate whether the subsequent word matched the object image.

All participants completed the Chinese (control condition, three blocks), English (three blocks), and switch (four blocks) conditions in the BCS task, which lasted 7–8 min and was presented on a 14-inch computer screen (resolution of 1,024 × 768) with a white background. Each block consisted of 10 trials. As shown in Figure 1, in one trial, the preschooler would be shown a brief (800 ms) picture stimulus and the corresponding language audio stimulus, and then asked to judge picture–audio agreement by pressing a mouse button as quickly as possible (maximum of 4,200 ms). The preschooler would get prompt audio feedback (500 ms) corresponding to his/her right or wrong response at the end of each trial. The formal experiment began when children’s accuracy exceeded 80% in the practice phase. Baseline rest lasting 60 s was conducted at the beginning of the formal experiment.

### The Task of Picture-Book Reading With the Additive Audio (PRA Task)

**FIGURE 2:**
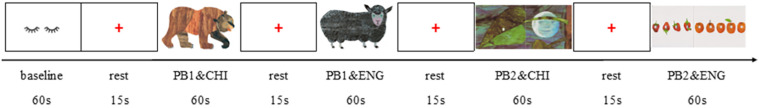
Brain imaging procedure for the picture-book reading with additive audio task. PB1 and CHI: each child was asked to read the first part of the picture book (*Bear*) and listen to the corresponding Chinese audio story. CHI, Chinese; ENG, English; PB1, the *Bear* picture book; PB2, the *Caterpillar* picture book.

We designed the PRA protocol to represent an ecological task state comparable to picture-book reading style in the real world. As shown in Figure 2, all preschoolers were asked to read two picture books of differing difficulty (adapted into wordless format), concomitant with a prompting audio story: *Brown Bear Brown Bear What Do You See* (picture book 1, PB1) and *The Very Hungry Caterpillar* (picture book 2, PB2). Specifically, the story of the picture book *Bear* involves just recognizing various animals, and the story is dominated by easily repeated sentences familiar to young children. In contrast, the story of the caterpillar’s growth from cocoon to butterfly is more complex, in terms of both story plot and sentence length. The reading sequence went from simple to complex, so the preschooler would not feel frustrated. Each picture book was divided into two parts, with one part accompanying the additive Chinese story audio and the other the English audio. The picture books, both written by internationally famous picture book writer Eric Carle, are in collage style and appropriate to read aloud. Both stories were recorded, without background music, by a kindergarten teacher. The audio sampling frequency was 44.1 KHz, and the sampling bit depth, 16 bits.

The study used E-prime (Psychology Software Tools Inc., Pittsburgh, PA, United States, Version 1.0) stimulation presentation software to present the experimental protocol. The run (Figure 2) began with a baseline white screen for 60 s and a red fixation point “+” that remained on the screen for 15 s. Afterward, the participants were asked to read the first part of PB1 (six pictures without text interpretation) and simultaneously listen to the Chinese audio story. After another 15 s rest (with the red fixation point “+” remaining), the second part of PB1 (the remaining six pictures) was displayed, and the corresponding English audio story was played. Following PB1, the participants completed the book-reading task for PB2, using the same process. All stimuli were displayed on a 14-inch computer screen (resolution of 1,024 × 768) with a white background.

Ensuring that children are familiar with experimental materials can help to ensure that differences in intra-brain activation represent the influence of language, rather than the children’s curiosity about the experimental materials. Thus, every participant was given the two experimental picture books and story audios 1 month before data collection and asked to read them with their parents at least once a week.

### NIRS Recording

**FIGURE 3:**
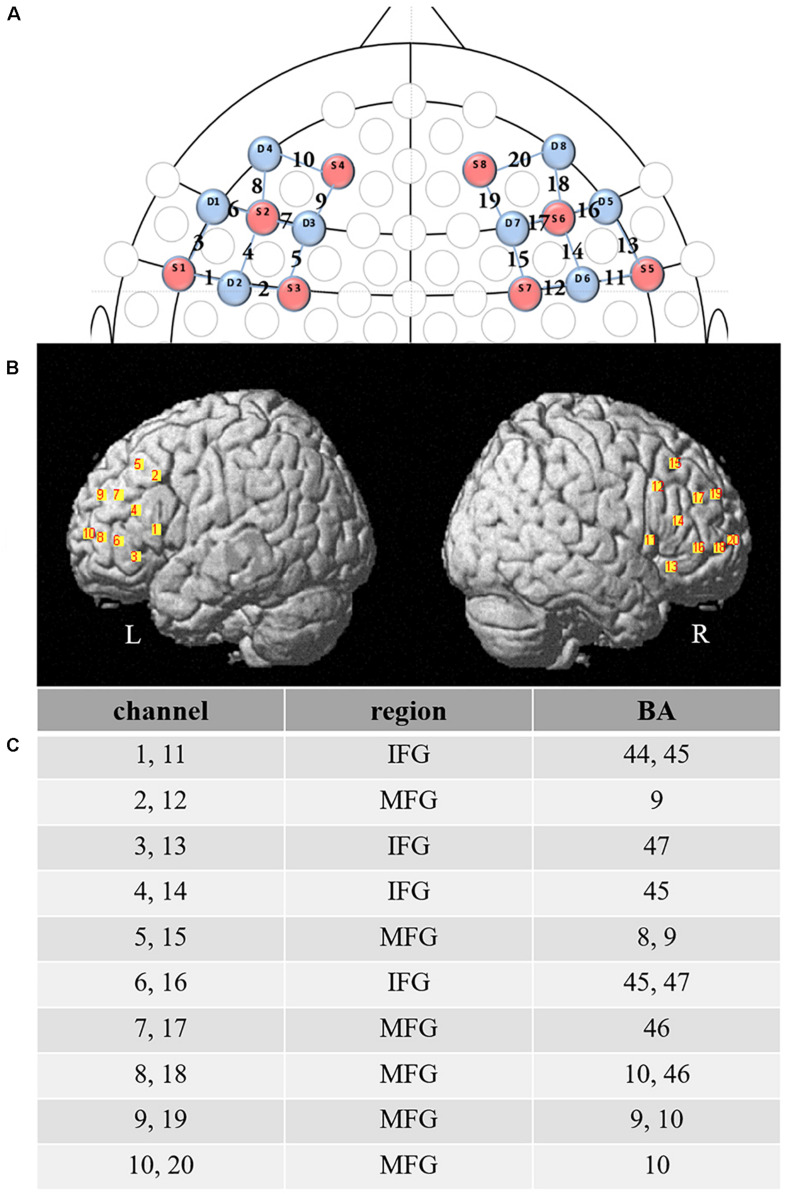
Functional near-infrared spectroscopy (fNIRS) channel and probe configuration. **(A)** 2-D map showing fNIRS configuration of probes (source – red, detector – blue) and channels between them. **(B)** Bilateral prefrontal cortex overlaid by the 20 channels. **(C)** Brodmann area (BA) maximally overlaid by each channel. L, the left brain; R, the right brain; IFG, inferior frontal gyrus; MFG, middle frontal gyrus.

We used the NIRSport portable near-infrared imager (NIRSport, NIRx Medical Technology LLC, Glen Head, NY, United States), with 630 and 850 nm wavelengths and a sampling rate of 7.81 Hz, to collect the prefrontal oxyhemoglobin (HbO) and deoxyhemoglobin (HbR) activation intensity changes in this study. The acquisition software used was NIRStar14.0. The fNIRS instrument consists of eight sources and eight detectors, with the inter-optode distance between transmitter and detector being set at 2.5 cm to form an effective measurement channel. The channel montage customized for this study included 20 channels that covered bilateral DLPFC and IFG (Figure 3). Per the 10–10 transcranial positioning system (Jurcak et al., 2007), the four reference detectors (1, 3, 7, and 5) were positioned at F7, F3, F4, and F8.

### Data Processing

All participants completed the entire task, and no participant was excluded from data analysis under the disqualified standard of 20% of the bad channel (i.e., four channels). The brain data were screened by Homer2 (Huppert et al., 2009), a software package based on MATLAB (The MathWorks, Inc., Natick, MA, United States), to convert optical density units, remove and correct motion artifacts, and make the band pass filter. First, the raw intensity data were converted into optical density units before undergoing quality detection steps using the channel artifact detection method (hmrMotionArtifactByChannel) and the Spline correction method (hmrMotionCorrectSpline) (Scholkmann et al., 2010). If any signal changes showed greater than the threshold of the standard deviation of 50 or amplifier class of 5 within 0.5 s and masked for an additional second, these artificial signal changes were corrected with a spline interpolation set to the 0.99 parameters. Then, a band pass filter with a cutoff frequency of from 0.01 to 0.2 Hz was applied. Previous studies have generally used frequency bands such as 0.01–0.1 Hz (Jiang et al., 2012) or 0.02–0.2 Hz (Jiang et al., 2015) to remove the high- and low-frequency noise, such as that associated with respiration (about 0.2–0.3 Hz) and cardiac pulsation (about 1 Hz). So, in our research, to remove high- and low-frequency noise and retain as much valid information as possible, we adopted the widest frequency band (0.01–0.2 Hz). Lastly, the filtered data were calculated for Hb concentration changes, per the modified Beer–Lambert Law (Delpy et al., 1988). We present the HbO signal in the study, owing to its higher sensitivity to blood flow changes (Strangman et al., 2002; Fu et al., 2014). Nevertheless, we conducted the Wilcoxon signed-rank test between HbO and HbR signal for verification of the validity of HbO results. As shown in Supplementary Figure S1 (the Hemodynamic response of channel 4 in the BCS task) and Supplementary Table S1 (Z scores of Wilcoxon test), children showed greater HbO than HbR signal.

### Statistical Analysis

All statistical tests were performed using IBM SPSS Statistics 25.0 (IBM Corporation, Armonk, NY, United States). The behavior data (accuracy and reaction time) and brain data (HbO signal) for the BCS task were subjected to a one-way repeated measure analysis of variance (ANOVA), with language comprehension (Chinese, English, and switching) as the within-subjects factor. We applied a 0.0025 (0.05/20) alpha level of significance in the multiple comparison test. Furthermore, we conducted a two-way repeated measure ANOVA [audio language types (Chinese versus English) × story difficulty (easy versus hard)] on the HbO data from the PRA task. The multiple comparisons analysis was subsequently tested using Bonferroni correction. Finally, Pearson’s correlation was performed to test the relationship between individual English competence and HbO activity for English story comprehension.

## Results

### Behavioral Performance

The EFL preschoolers’ Chinese vocabulary competence (see Table 1) was not different from that of the typical Chinese monolinguals [*t*_(PPVT)_ = 0.85, *p* > 0.05; *t*_(EVT)_ = 0.35, *p* > 0.05] who participated in our previous study [*M*_(PPVT)_ = 74.35 ± 15.51; *M*_(EVT)_ = 73.05 ± 14.81, *n* = 66] (Li et al., 2019), indicating that the EFL preschoolers’ Chinese language competence was on a normal development trend. However, the children’s English abilities were inferior to their Chinese abilities, based on their accuracy and reaction time in the BCS tasks (see Table 1); multiple comparisons analysis using Bonferroni correction further revealed accuracy (*p* < 0.001) and reaction time (*p* < 0.001) advantages in their CC over their EC. Moreover, the children showed weak performance in the condition of BS, compared to their CC accuracy (*p* < 0.001) and reaction time (*p* < 0.001). Thus, the EFL preschoolers were Chinese–English unbalanced bilinguals.

**TABLE 1:** Participants’ language performance.

Measures (*n* = 26)	*M*	*SD*
LANGUAGE TEST		
PPVT (Chinese)	76.77	12.83
EVT (Chinese)	74.85	13.89
PLS_AC (English)	12.12	5.82
PLS_EC (English)	14.62	4.22
BCS ACCURACY (%)		
Chinese comprehension	92.33	8.54
English comprehension	78.00	12.71
Bilingual switching	78.17	8.72
BCS REACTION TIME (ms)		
Chinese comprehension	1, 553.99	286.03
English comprehension	1, 791.35	253.32
Bilingual switching	1, 766.24	206.69

*PPVT, Peabody Picture Vocabulary Test; EVT, Expressive Vocabulary Test; PLS, Preschool Language Scale; AC, auditory comprehension; EC, expressive communication; BCS, bilingual comprehension switch task.*

### Prefrontal Activation in Response to BCS Task

**FIGURE 4:**
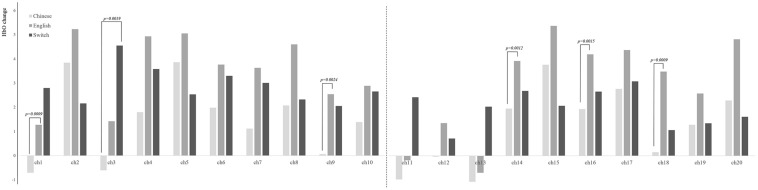
Brain activation for each condition on 20 channels. Chinese, Chinese comprehension; English, English comprehension; Switch, bilingual switching condition.

After controlling the variable of children’s months of age, repeated measure analyses of the three conditions of the BSC task showed that the condition of EC displayed greater activation than did CC on channels 1, 9, 14, 16, and 18 using a 0.0025 (0.05/20) alpha level of significance (20 channels) (see Figure 4), covered with bilateral IFG and DLPFC. In addition, compared with the condition of CC, the condition of BS was discovered to have higher activation on the left IFG (channel 3) (*p* = 0.0019). As shown in Supplementary Figure S2, children decreased their level of HbR activity on the PFC in the conditions of EC and BS (*p*s < 0.0025), compared with the condition of CC.

### Effect of Story Audio on Prefrontal Activation in PRA Task

**FIGURE 5:**
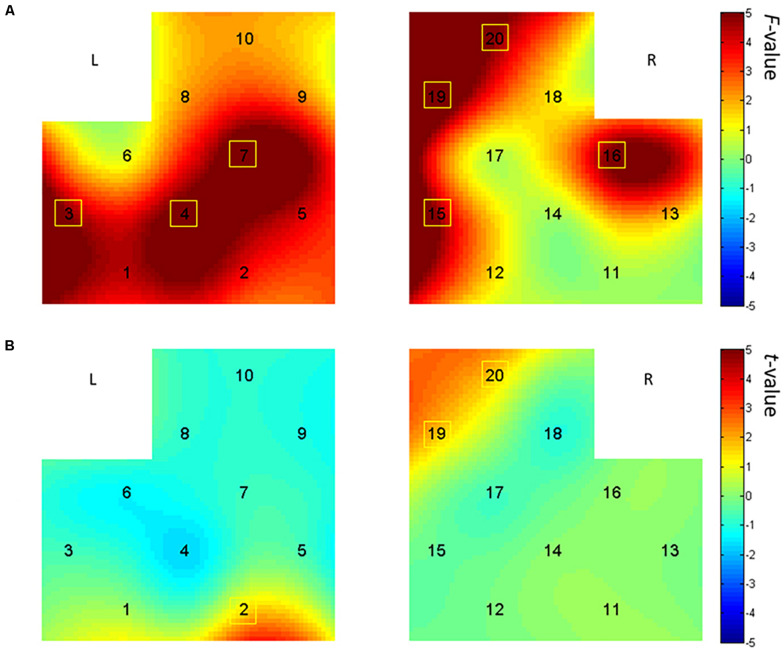
Brain activation maps in the picture-book reading with additive audio (PRA) task. **(A)** Main effect analysis results of language type displayed by the interpolated *F*-values, with brighter color representing higher activation in the English PRA condition than in the Chinese PRA condition. **(B)** In the processing of picture-book reading with English audio, the preschoolers showed greater brain activation on channels 2, 19, and 20 in the more difficult picture-book reading (PB2) than in the easier one (PB1). L, left hemisphere; R, right hemisphere.

Based on the analysis of activation differences in dual language vocabulary comprehension, we further explored the effect of story comprehension on EFL children’s prefrontal activation, using the bilingual picture-book story. PB1 (*Bear*) is easier than PB2 (*Caterpillar*) in terms of both sentence length and story plot. We conducted a two-way ANOVA [audio language types (Chinese vs. English) × story difficulty (PB1 vs. PB2)] on HbO in all 20 channels and discovered (from Figure 5A) main effects on audio language type in bilateral IFG (Ch. 3, 4, 16) and middle frontal gyrus (MFG) (Ch. 7, 15, 19, 20), with no significant main effect on story difficulty type. The results reveal significantly higher activation in English PRA than Chinese PRA on those channels [Ch. 3: *F*(1,25) = 6.58, *p* = 0.017, partial η^2^ = 0.21; Ch. 4: *F*(1,25) = 5.77, *p* = 0.024, partial η^2^ = 0.19; Ch. 7: *F*(1,24) = 6.16, *p* = 0.020, partial η^2^ = 0.20; Ch. 15: *F*(1,24) = 7.05, *p* = 0.014, partial η^2^ = 0.23; Ch. 16: *F*(1,25) = 5.58, *p* = 0.026, partial η^2^ = 0.18; Ch. 19: *F*(1,23) = 9.57, *p* = 0.005, partial η^2^ = 0.29; Ch. 20: *F*(1,25) = 4.54, *p* = 0.043, partial η^2^ = 0.15].

Besides the significant main effect of audio language type, the interaction effect between language type and story difficulty was significant in channel 2 [*F*(1,25) = 9.51, *p* = 0.005, partial η^2^ = 0.28], channel 19 [*F*(1,23) = 8.35, *p* = 0.008, partial η^2^ = 0.27], and channel 20 [*F*(1,25) = 3.38, *p* = 0.018, partial η^2^ = 0.20]. Multiple comparisons analysis using Bonferroni adjustment revealed higher activation in the more difficult picture-book reading (PB2, with longer sentences and more complex plot) than in the easier picture-book reading condition in the English PRA task in the MFG regions [*p*(Ch. 2) = 0.002; *p*(Ch. 19) = 0.016; *p*(Ch. 20) = 0.022]. These results were similar to the outcomes exhibited in Figure 5B by the pairwise sample *t*-test analysis between the more difficult PB2 and the easier PB1 reading in the English PRA tasks [Ch. 2: *t*(26) = 3.40, *p* = 0.002; Ch. 19: *t*(24) = 2.61, *p* = 0.016; Ch. 20: *t*(26) = 2.441, *p* = 0.022].

### Relationship Between the Brain Activation of English PRA Task and English Competence

Pearson’s correlation analyses revealed that preschoolers’ PLS-AC scores significantly correlated with brain activation for PB1 EC in the PRA task on channel 6 [*r*(26) = 0.54, *p* = 0.004], channel 7 [*r*(25) = 0.51, *p* = 0.009], channel 9 [*r*(26) = 0.46, *p* = 0.018], and channel 15 [*r*(25) = 0.44, *p* = 0.028], and the activation of PB2 EC on channel 6 [*r*(26) = 0.42, *p* = 0.03] and channel 19 [*r*(24) = 0.43, *p* = 0.036]. Moreover, children’s PLS-EC scores only showed correlation with activation of PB2 (without PB1) EC on channel 1 [*r*(24) = 0.44, *p* = 0.032] and channel 19 [*r*(24) = 0.43, *p* = 0.035]. In conclusion, children’s English competence, especially in terms of their AC ability, was correlated with activation of the left IFG (Ch. 6) and bilateral DLPFC (Ch. 7, 9, 15, 19) in the English PRA tasks.

## Discussion

This present study was designed to explore whether L2 processing (from auditory word recognition to auditory story comprehension) affected the functionality of EFL preschoolers’ cortical brain regions for domain-general cognitive control and language processing. The first aim was to observe differences in bilingual preschoolers’ prefrontal activation in three processes of word comprehension, including different cognitive control demands. In addition, we examined the effect of picture-book reading with additive dual language audio on EFL preschoolers’ prefrontal activation. Lastly, the relationship between individual differences in English competence and brain activation was investigated. The results and implications are discussed as follows.

### Brain Activation Pattern for Word Comprehension

According to joint activation theory, both languages will be activated when bilinguals use either language, and the competition between dual languages may be an inherent process (Bialystok, 2011; Kroll et al., 2015). On the other hand, the inhibitory control theory model (Green, 1998; Giezen et al., 2015) claims that the conflict is resolved by the suppression of the non-target language competitor, with the selection involving the domain-general executive control system in charge of the PFC (Miller and Cohen, 2001; Ridderinkhof et al., 2004). An important assumption of this model is that the proficiency of the suppressed language determines the magnitude of inhibition; thus it can be seen that higher inhibition demands for proficient Chinese (non-target language) would be invoked when EFL preschoolers handled the English (target language) condition in our BCS task.

Consequently, the inhibitory control model perfectly explains our finding that there was greater prefrontal activation (IFG and DLPFC) in the process of understanding English words than in the Chinese context in this BCS task. Moreover, in many studies, unbalanced bilinguals’ behavior performance when handling their native language outperformed their responses to L2 (for example, Christoffels et al., 2007; Ivanova and Costa, 2008). Based on their accuracy and reaction time in the BCS task, EFL preschoolers should also be regarded as unbalanced bilinguals. Thus, the inhibition of their dominant Chinese inevitably engaged more prefrontal resources in charge of the cognitive control system in the process of understanding non-proficient English stimulus words.

We also found that the condition of bilingual comprehension switching caused greater activation on the left IFG than did the control condition of CC. This result is in line with those shown in many prior studies using a language production paradigm, such as picture or digit naming tasks. In a quantitative meta-analysis of functional neuroimaging studies, Luk et al. (2012) compared BS conditions with baseline conditions of using a single language and summarized that eight brain regions – including the left IFG – showed significant and reliable activation. Further evidence reported by Anderson et al. (2018) supports a functional overlap in bilinguals’ left IFG in verbal and non-verbal switching tasks. Arredondo’s group (2017) also found greater left frontal lobe activation during a non-verbal attention task among bilingual children compared with monolinguals.

A neuroimaging study using the lexical selection paradigm also showed that bilinguals engaged frontal regions and the caudate and putamen, when presented with between-language competition (Marian et al., 2017). However, our result of recruiting a lot of left PFC resources in the language switching condition is inconsistent with the recent fNIRS evidence that bilingual school-aged children (7–10 years old) showed greater activation in posterior temporo-parietal regions compared with monolingual children in a lexical selection task (Arredondo et al., 2019a). One important reason may be that our participants are 5- to 7-year-old EFL children who have insufficient bilingual experience in the English immersion program. Between the ages of 7 and 10, children’s neural organization for language processing gradually becomes more adult-like in the left language neural network, with the differentiated system for language function of phonological, semantical, and syntactic processing (Skeide et al., 2014). The abundant bilingual exposure in this age range may have reconstructed the neural network on the formation of attention-demanding word identification processes to a large extent. The BAPSS theory and Neuroemergentism framework claim that the shift of recruitment from frontal to posterior and subcortical regions is modulated with L2 exposure and learning time (Grundy et al., 2017; Hernandez et al., 2018). In other words, young children who are on the way to learning English perhaps undergo the early stages of neural specialization. Given the greater cognitive control demands, it is not surprising that greater activation was shown in the prefrontal brain in the BS condition of this BCS task, especially the left IFG, which is associated with monitoring and conflict resolution.

Taken together, in accordance with the bilingual language control system (Calabria et al., 2018), increasing demands on language control in the two contexts (English using and BS) enhanced the recruitment of prefrontal networks responsible for domain-general cognitive control. The model of the adaptive control hypothesis (Green and Abutalebi, 2013; Abutalebi and Green, 2016) of bilingual processing further suggests that the left PFC is responsible for language selection.

### Brain Activation Pattern for Auditory Story Comprehension

Previous bilingual neuroimaging studies always measured an individual’s brain activity in association with relatively transient language stimuli, such as word processing (e.g., picture naming, Chen et al., 2020) or morpho-syntactic form contrasts (e.g., grammaticality judgment, Arredondo et al., 2019b). This study investigated the effect of L2 processing on children’s brain responses in a more ecological and natural situation, hearing sentence stimuli full of prosodic features embedded in the process of picture-book reading. As mentioned earlier, there is no doubt that exploration of the effect of natural L2 learning on children’s brain development contributes to the debate over bilingual advantage.

The benefits of picture-book reading in cognition, social emotion, language, and neural structure are well documented in children (for instance, Hutton et al., 2015; U.S. Department of Education, 2015). Moreover, relative to a single auditory story without picture book, PRA could increase the functional connectivity between visual perception, default mode, and cerebellar networks (Hutton et al., 2020). Therefore, the functional connectivity pattern of PRA may be an efficiently functional model for supporting higher-order skill (Bullmore and Sporns, 2012).

Thus, the PRA method would be an effective way for children to learn a new language, given Horowitz-Kraus et al.’s (2013) evidence that auditory narrative comprehension is a higher-order skill associated with executive function and linguistic skills. In our story-processing study, as expected, the findings indicate that English PRA caused greater activation than Chinese PRA in the bilateral IFG and MFG, which is in line with the discovery of the BCS task. It might be speculated that English auditory narrative comprehension in the PRA task was a complex version of English word comprehension of the BCS task. Consequently, it was not surprising that the non-balanced bilingual preschoolers needed to inhibit their native Chinese language activation while understanding the English story. It relied on basic networks of visual imagination and executive function in the processing of EC (Horowitz-Kraus et al., 2013; Hutton et al., 2015) and inevitably recruited the PFC region (Miller and Cohen, 2001), especially the DLPFC (Ridderinkhof et al., 2004), the hub region of domain-general cognitive control.

This study has further demonstrated that the difficulty of the auditory story influenced the recruitment of bilateral MFG networks in the English PRA condition. More precisely, longer sentences or a more complex auditory story might generate more MFG brain activation in the process of comprehending the English story. In a recent study, Sulpizio et al. (2020b) found that the larger involvement of dorsal-stream PFC resources in difficult rather than easy conditions could be related to the additional demands of phonological computation and cognitive decoding in L2. Thus, the more difficult auditory story engaged more brain networks for language processing and cognitive processing, to a certain extent. Nevertheless, given Vygotsky’s zone of proximal development theory, we suggest that increasing L2 difficulty in the range of children’s English competence contributes to the bilingual advantage.

### The Relationship Between Brain Activity and Individual Ability

It is worth noting that we observed a positive correlation between children’s English competence and PFC activation in the process of their comprehending the English story. EFL children’s better PLS assessment performance, especially in terms of AC, showed greater activation of their left IFG and bilateral DLPFC. One possible reason for this is that increased L2 proficiency has gradually reshaped the bilinguals’ brain networks for language control and even domain-general control to some extent. Young children, especially, tend to rely heavily on PFC resources for bilingual control tasks [see more on the BAPSS theory in Grundy et al. (2017)]. Robust functional imaging evidence shows that the left IFG and DLPFC play important roles in those control processes (Calabria et al., 2018). Furthermore, many structure imaging studies have associated higher L2 proficiency with greater structural density in the brain regions supporting executive control and language processing (Mechelli et al., 2004; Hosoda et al., 2013; Pliatsikas et al., 2015; Del Maschio et al., 2018). For example, Gullifer et al. (2018) recently associated the function connection of language- and control-related networks with a balanced bilingual proficiency, quantified by language entropy.

To summarize, the PRA method might greatly help EFL preschoolers improve their English proficiency (Sun et al., 2016). At the same time, our findings provide further evidence that individual English proficiency could modulate bilinguals’ efficiency in brain function reorganization of language control and domain-general executive control. Therefore, we suggest that picture-book reading with L2 audio might contribute to generating a bilingual advantage and molding the bilingual brain. However, as the correlation was acquired from a small sample, caution should be exercised.

### Limitations and Future Directions

The results of this ecological neuroimaging study demonstrate the differences in children’s brain activation during dual language processing (comprehending auditory vocabulary and story) and the modulation of brain activity intensity by both preschoolers’ English competence and story difficulty. The study contributes to answering questions about whether and how picture-book reading enhances bilingual advantage. However, as there was a lack of participants from low-income families, all the EFL children came from middle- and high-SES families. Because SES is closely related to children’s cognition (Lawson et al., 2018), language (Levine et al., 2020), and brain development (Farah, 2017), the small sample size confines the generalizability of the findings to some extent; future studies with more participants (including a low-SES group) should be conducted. Furthermore, one recent study associated with the bilingual control mechanism has revealed that language control for perception and for production might involve different neural circuitry (Blanco-Elorrieta and Pylkkänen, 2016). Future ecological research should consider the tasks of language production and comprehension together, especially for non-balanced bilingual children. Finally, given the latest direction in bilingualism study – i.e., that bilingualism is not a categorical label (Luk and Bialystok, 2013) and should be regarded as a gradient or spectrum of the experience-based dynamic process (Hernandez et al., 2018; DeLuca et al., 2019, 2020; Pliatsikas, 2019; Pliatsikas et al., 2019; Sulpizio et al., 2020a) – future studies on the effects of bilingualism on children’s brains should consider complex variables, ecological validity, and tracking paradigm.

## Data Availability Statement

The raw data supporting the conclusions of this article will be made available by the authors, without undue reservation.

## Ethics Statement

The studies involving human participants were reviewed and approved by the East China Normal University Institutional Review Board on Human Research Protection. Written informed consent to participate in this study was provided by the participants’ legal guardian/next of kin.

## Author Contributions

CL, JZ, and DY designed the experiments. CL, KD, and MZ analyzed the data and wrote the first draft. LZ proofread and discussed the manuscript. All authors contributed to the article and approved the submitted version.

## Conflict of Interest

The authors declare that the research was conducted in the absence of any commercial or financial relationships that could be construed as a potential conflict of interest.
