# Supplementary material for: Effect of Picture-Book Reading With Additive Audio on Bilingual Preschoolers’ Prefrontal Activation: A Naturalistic Functional Near-Infrared Spectroscopy Study
Source: Front Psychol. 2020 Aug 5;11:1939. doi: 10.3389/fpsyg.2020.01939 (PMC7419625; doi:10.3389/fpsyg.2020.01939)
Supplement: Supplementary file 1 [file Image_1.PDF]

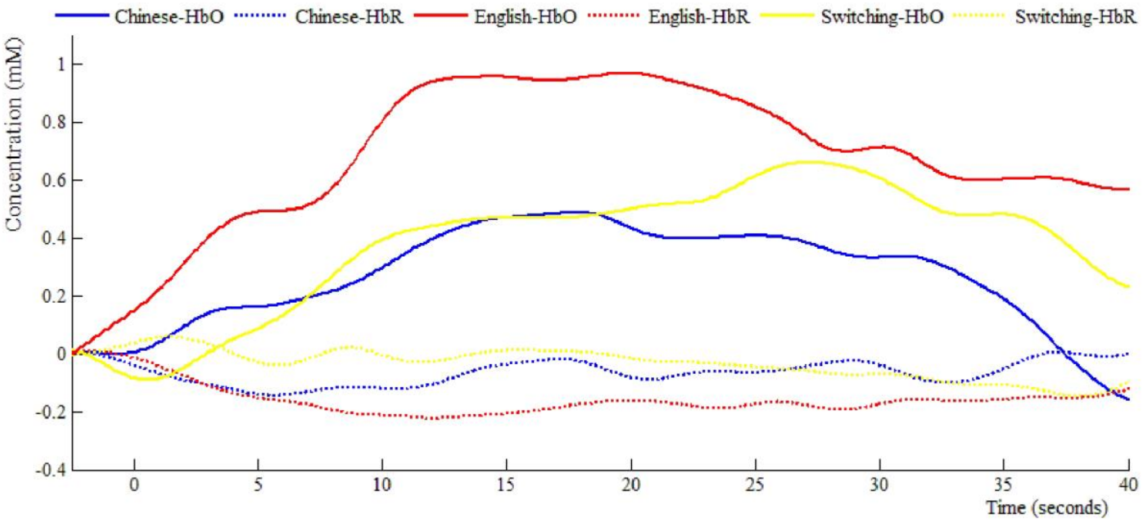

Supplementary Figure 1. Hemodynamic response of channel 4 in the BCS task. Children showed greater HbO than HbR signal in each condition of BCS task.
