# Supplementary material for: Effect of Picture-Book Reading With Additive Audio on Bilingual Preschoolers’ Prefrontal Activation: A Naturalistic Functional Near-Infrared Spectroscopy Study
Source: Front Psychol. 2020 Aug 5;11:1939. doi: 10.3389/fpsyg.2020.01939 (PMC7419625; doi:10.3389/fpsyg.2020.01939)
Supplement: Supplementary file 2 [file Image_2.PDF]

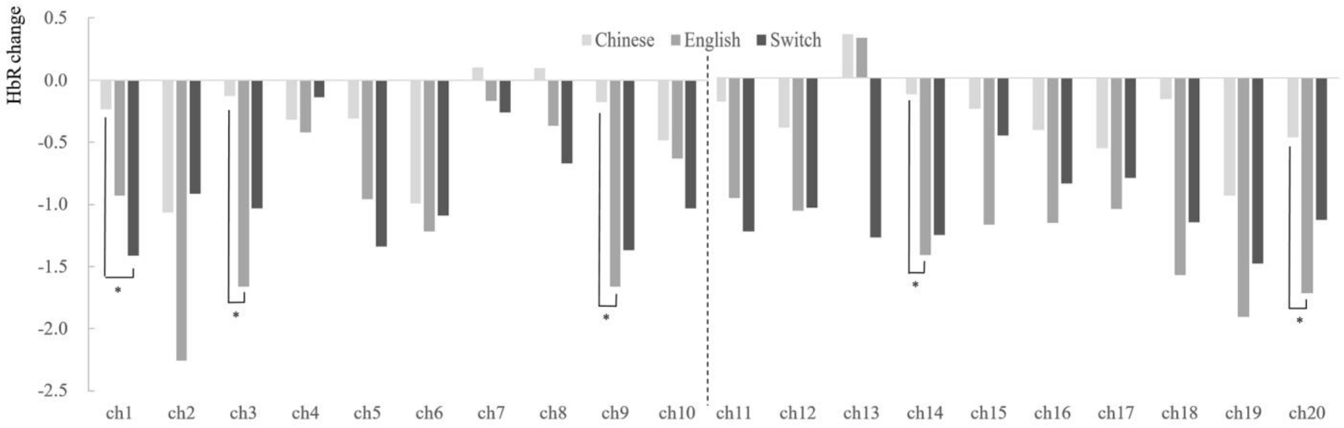

Supplementary Figure 2. HbR analysis for each condition on 20 channels. Chinese: Chinese comprehension (CC). English: English comprehension (EC). Switch: bilingual switching condition (BS). * $p<0.0025$. The condition of EC decreased the robust level of HbR activity on bilateral IFG and DLPFC more than did the condition of CC, while the condition of BS reduced the HbR concentration on left IFG.
