# Supplementary material for: Effect of Picture-Book Reading With Additive Audio on Bilingual Preschoolers’ Prefrontal Activation: A Naturalistic Functional Near-Infrared Spectroscopy Study
Source: Front Psychol. 2020 Aug 5;11:1939. doi: 10.3389/fpsyg.2020.01939 (PMC7419625; doi:10.3389/fpsyg.2020.01939)
Supplement: Supplementary file 3 [file Table_1.DOCX]

**Supplementary Table 1**

The Wilcoxon test was adopted to test the difference between HbO and HbR in the BCS task. The positive values (Z scores) mean HbO inceased more than HbR in the task. Results showed that children showed significantly greater HbO than HbR signal in the BCS task(**p*<0.05).

|  | ***ch1*** | ***ch2*** | ***ch3*** | ***ch4*** | ***ch5*** | ***ch6*** | ***ch7*** | ***ch8*** | ***ch9*** | ***ch10*** |
| --- | --- | --- | --- | --- | --- | --- | --- | --- | --- | --- |
| **Chinese** | -0.040 | 3.551* | -0.654 | 2.335* | 2.680* | 3.434* | 1.242* | 1.895 | 0.121 | 1.965* |
| **English** | 2.617* | 3.682* | 2.243* | 2.536* | 2.810* | 3.007* | 2.287* | 3.246* | 2.979* | 2.013* |
| **Switching** | 2.978* | 1.982* | 3.288* | 2.496* | 3.202* | 2.771* | 3.290* | 3.242* | 3.663* | 2.344* |
|  | ***ch11*** | ***ch12*** | ***ch13*** | ***ch14*** | ***ch15*** | ***ch16*** | ***ch17*** | ***ch18*** | ***ch19*** | ***ch20*** |
| **Chinese** | -0.501 | 0.564 | -0.909 | 2.021* | 2.617* | 2.157* | 3.260* | -0.644 | 2.434* | 2.777* |
| **English** | 0.240 | 2.214* | -0.625 | 3.155* | 3.623* | 3.028* | 3.140* | 3.100* | 3.465* | 3.784* |
| **Switching** | 2.548* | 2.013* | 2.329* | 3.725* | 1.972* | 2.679* | 3.462* | 2.158* | 2.048* | 2.133* |
